# The regulation of plant cell wall organisation under salt stress

**DOI:** 10.3389/fpls.2023.1118313

**Published:** 2023-03-10

**Authors:** Siarhei A. Dabravolski, Stanislav V. Isayenkov

**Affiliations:** ^1^ Department of Biotechnology Engineering, Braude Academic College of Engineering, Karmiel, Israel; ^2^ Department of Plant Food Products and Biofortification, Institute of Food Biotechnology and Genomics, National Academy of Science (NAS) of Ukraine, Kyiv, Ukraine

**Keywords:** salinity stress, stress tolerance, regulatory mechanisms, cell wall composition, transcriptional regulation, root barriers of salt transport

## Abstract

Plant cell wall biosynthesis is a complex and tightly regulated process. The composition and the structure of the cell wall should have a certain level of plasticity to ensure dynamic changes upon encountering environmental stresses or to fulfil the demand of the rapidly growing cells. The status of the cell wall is constantly monitored to facilitate optimal growth through the activation of appropriate stress response mechanisms. Salt stress can severely damage plant cell walls and disrupt the normal growth and development of plants, greatly reducing productivity and yield. Plants respond to salt stress and cope with the resulting damage by altering the synthesis and deposition of the main cell wall components to prevent water loss and decrease the transport of surplus ions into the plant. Such cell wall modifications affect biosynthesis and deposition of the main cell wall components: cellulose, pectins, hemicelluloses, lignin, and suberin. In this review, we highlight the roles of cell wall components in salt stress tolerance and the regulatory mechanisms underlying their maintenance under salt stress conditions.

## 1 Introduction

High salinity threatens more than 20% of irrigated lands worldwide, and this area is dramatically increasing every year, greatly affecting plant growth and yield through sodium accumulation-mediated osmotic and toxicity stresses (Singh, 2021). During their evolution, plants have developed various strategies to cope with the high soil salinity problem, such as osmotic and metabolic adjustment, normalisation of ion levels and Reactive oxygen species (ROS) balance, salts extrusion or safe accumulation and storage, hormonal and epigenetic re-arrangements, life cycle shortening, or re-schedule salt-sensitive developmental stages (Zhao et al., 2020; Rahman et al., 2022).

Despite intensive research, the exact mechanism by which Na^+^ and Cl^-^ enter the roots is still unknown. As was shown in several species, up to 50% of Na^+^ and Cl^-^ of total uptake translocate *via* apoplast, suggesting the importance of salt transport route *via* cell walls during high salinity. The symplastic uptake is mediated *via* various channels and transporters, such as NSCCs (nonselective cation channels) and GLRs (glutamate receptor–like channels), HKTs (high-affinity K^+^ transporters), PIPs (plasma membrane intrinsic proteins), LCTs (low-affinity cation transporters), AKT (Arabidopsis K^+^ Transporter) and KAT (K+ channel in Arabidopsis Thaliana), CCCs (cation-coupled chloride cotransporters) and others [reviewed in (Isayenkov and Maathuis, 2019)]. Similarly, several sensors and receptors could recognise osmotic and ionic stresses and initiate signal transduction and adaptation responses in plants. For example, high salt concentration alters the balance of cell wall ions, which is sensed by sensors or receptors [such as RLKs (receptor-like kinases), GIPC (glycosyl inositol phosphorylceramide), and FER (Feronia)] and activates specialised signaling pathways to normalise the ions balance (such as the SOS pathway). The disbalance of outside/inside ions is sensed by osmosensors [such as HPKs (histidine protein kinases), NSCCs, BON (BONZAI1)], which initiate ions uptake and synthesis of osmolytes (such as proline) to normalise the osmotic homeostasis. Further details of the current knowledge of the plant’s osmotic and Na^+^ sensors could be found in the recent review (Wang et al., 2022). Additionally, many plant hormones [not only ABA (abscisic acid) and cytokinin but also auxin, salicylic acid, gibberellin, ethylene, and others] are involved in the regulation of the defence system and growth adaptation under salt stress (Yu et al., 2020).

**Figure 1:**
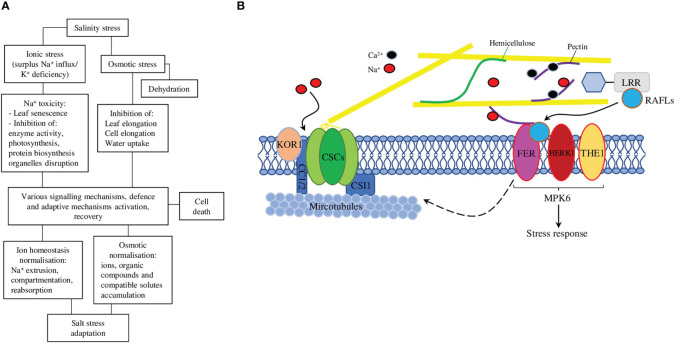
**(A)** A schematic representation of the effects of salt stress on plants and the corresponding responses that plants use to adapt and survive these detrimental effects. **(B)** Salt stress exposure affects cortical microtubule polymerisation and CSC (cellulose synthase complex) assembly. At the recovery stage, FER (FERONIA) regulates cortical microtubule reassembly and the relocation of CSCs to the plasma membrane to synthesise cellulose, thus enhancing plant adaptation to salt stress. The cell wall sensor FER-LRRs-RALF module works in association with HERK1 (HERKULES1) and THE1 (THESEUS1) to perceive salinity through the perturbation in pectins and acts *via* MPK6 (mitogen-activated protein kinase 6) to initiate a salt stress response. The dashed arrow represents in-direct regulation, solid arrows—direct regulations/interactions.

Salt stress directly and accompanying ionic, osmotic, and oxidative stresses impair ion homeostasis and damage biomolecules, thus interrupting normal physiological processes and preventing plant normal growth, development, and sometimes even survival (**Figure 1A**) (van Zelm et al., 2020). The plant cell wall, however, is the first barrier between cell content and external salt. Plant cell walls consist of polysaccharides, various structural proteins, and fatty acid–derived compounds (such as cellulose, hemicelluloses, pectins, lignin, and suberin), which are crucial for plant growth, development and protection from adverse environmental influences (both biotic and abiotic) (Lampugnani et al., 2018). The primary cell wall is a thin layer composed of the polysaccharides cellulose, hemicelluloses (mostly xyloglucan), and pectin; it is permeable to small molecules, flexible, and extensible, thus facilitating the cell’s growth. The secondary cell wall is a thick layer formed inside the primary cell wall in some cell types and made of cellulose, hemicellulose (mostly xylan) and lignin, which make it stiff and waterproof (Zhang et al., 2021). The negative charge of the cell wall is physiologically important, because it facilitates the reversible binding of Ca^2+^, which is used to strengthen the cell wall *via* pectins cross-linking. However, under high salt concentrations, the surplus of Na^+^ could replace Ca^2+^, thus interrupting normal pectins cross-linking and cell elongation (**Figure 1B**) (Hocq et al., 2017). At the same time, the amount of different negatively charged polymers able to bind Ca^2+^ in the cell wall stronger could be up-regulated, thus preventing various high Na^+^-associated toxic effects on the cell wall (Edmond Ghanem et al., 2010).

**Figure 2:**
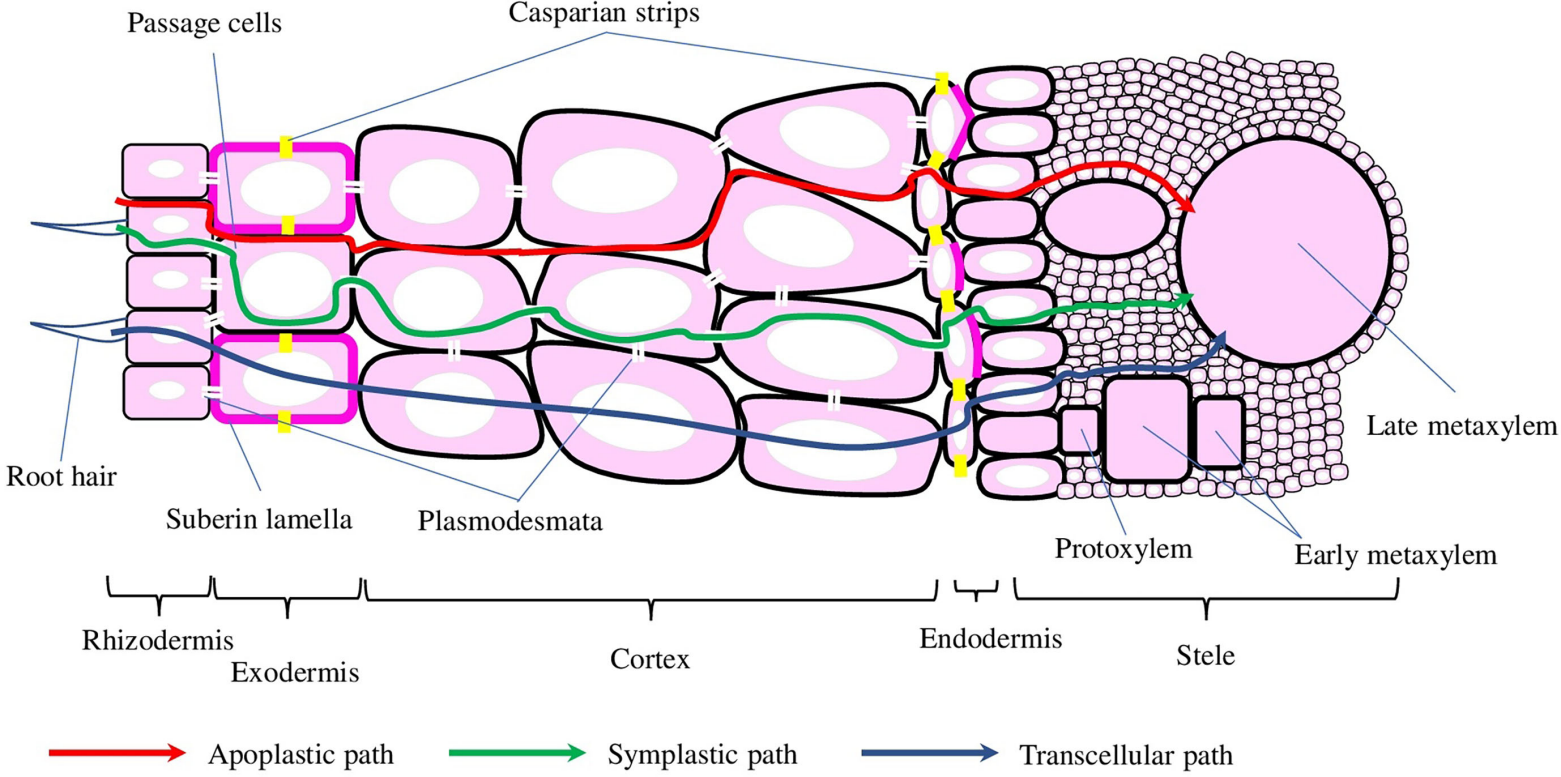
Schematic representation of the cross-root transport mechanisms and root cross-section. Water and solutes can be transported into the stele *via* apoplastic, symplastic, and transcellular mechanisms, depicted with red, green and blue arrows, respectively. Casparian strips and suberin lamellae (depicted with yellow and magenta colours, respectively) in the endo- and exodermis interrupt water and solute transport into the stele.

The transport of water and solutes across plant roots can occur *via* apoplast, symplast, transcellular, or a combination of these three (**Figure 2**). The endodermis is the first diffusion barrier, preventing radial apoplastic flow. Exodermis is the second barrier developed by some plant species located beneath the epidermis and can consist of one or several layers with increased amounts of suberin, lignin, proteins, and carbohydrates, which would depend on the species, variety, developmental stage, or environmental conditions (Kim et al., 2018). Suberin is the major component, making suberised cells almost impermeable to water and providing a critical protective layer preventing water and solutes loss, and the entrance of pathogens. Suberin biosynthesis, assembly, and deposition processes are tightly orchestrated by environmental and developmental stimuli, which involve a wide range of TFs, hormones, and enzymes. The enzymes that are responsible for the synthesis of phenolic and aliphatic monomers and their subsequent esterification, deposition, and assembly (Woolfson et al., 2022) are regulated by TFs (not only of MYB family but also NAC and WRKY) and hormones (mostly by abscisic acid, but jasmonic acid, salicylic acid, and ethylene are also involved) (Nomberg et al., 2022).

The development of endodermis is generally divided into three stages: (1) the deposition of a ring of lignin around the endodermal cells, which resulted in the formation of the Casparian strips (or bands) (CS). Despite CS preventing water and solutes diffusion, endodermal cells on stage 1 still could contribute to both symplastic and trans-cellular flows (Naseer et al., 2012). (2) The deposition of suberin throughout the cell wall greatly slows down nutrient uptake, while preserving symplastic connection with neighbour cells through plasmodesmata (Sager and Lee, 2014). (3) On the final stage, the cell wall is extensively thickened with the deposition of cellulose in a stele-ward direction (Kim et al., 2018). Even though the development of CS and suberin deposition happens at the same time point, the general features of exodermis are similar to the endodermis (Serra and Geldner, 2022).

The exact composition and structure of plant cell wall components are dynamically modulated, thus allowing cells to execute the optimal growth programme and timely respond to different stresses (Rui and Dinneny, 2020). The common salt stress response includes lignin accumulation, disruption of pectins cross-linking, reduction of cellulose content, and enhancement of antioxidant defence. Furthermore, plants defective in cell wall biosynthesis have demonstrated increased sensitivity to salt stress, thus confirming the importance of cell wall integrity maintenance for salt stress adaptation Liu et al., 2021). Further in this review, we focus on the recent discoveries of the molecular mechanisms connecting cell wall integrity and salt tolerance in plants.

## 2 Role of cell wall component in plant salt tolerance

### 2.1 Cellulose

The process of cellulose biosynthesis is complex and tightly regulated, and it is interrupted by salt stress on multiple levels and resulted in growth defects/inhibition, anatomical abnormalities, and hypersensitivity to salt stress. Several transcription factors, epigenetic regulatory mechanisms, and hormones, have been shown to affect the cellulose biosynthesis process upon salt stress perception (**Table 1**). However, salt-mediated modulation of the cellulose biosynthesis process should be always considered in close association with other metabolic and physiological responses caused by salt stress. Further investigation and understanding of the exact molecular mechanisms of every cellulose biosynthesis-associated genes/proteins involved in salt stress tolerance development would promote its practical application in creating salt-tolerant varieties of different plant species.

**Table 1:** List of genes associated with cell wall biosynthesis and salt tolerance.

Species	Gene	Phenotype under salt stress	gene regulation	References
*Arabidopsis thaliana*	*CESA1* and *CESA6*	c*esA1* and c*esA6* plants are salt sensitive, with severe root tip swelling, reduced cellulose contents and root elongation	–	(Zhang et al., 2016)
	*CSI1*	*csi1* plants are salt stress hyper-sensitive	–	
	*KOR1*	*kor1* plants are salt stress-sensitive, with marked cellulose deficiency and retarded growth	–	(Nagashima et al., 2020)
	*HSFA7b*	*HSFA7b* OE plants had increased proline and soluble sugar levels, and salt-stress tolerance.	Ex: *AtP5CS1* and *AtP5CS2* up-regulated LF: *AtP5CS1* and *AtP5CS2* down-regulated. AtHSFA7b down-regulates 31 cellulose biosynthesis-related genes (including *CESA8*, *CSLG1*, *CSLG2*, and *CSLA9)* and induces other TFs (bHLH, NAC061, NAC036, NAC090, WRKY38, and ZFP2)	(Zang et al., 2019)
	*GCN5*	*gcn5* plants have reduced cellulose content, and exhibit severe inhibition of growth and cell wall anomaly	GCN5 regulates *CTL1*, *PGX3*, *MYB54*	(Zheng et al., 2019)
	*XTH30*	*XTH30* Ex plants hypersensitive to salt stress; *xth30* plants are salt tolerant, have lower Na^+^ accumulation in shoot and H_2_O_2_ content		(Yan et al., 2019).
	*XTH19* and *XTH23*	*xth23* and *xth19/xth23* plants are additively sensitive to salt stress; *BES1* Ex increased salt tolerance in WT and improved salt tolerance of *xth19/xth23*.	BES1 regulates *XTH19* and *XTH23*	(Xu et al., 2020)
	*PME31*	*PME31* LF plants are salt-stress hypersensitive to salt stress;	LF: *DREB2A, RD29A* and *RD29B* down-regulated	(Yan et al., 2018)
	*MYB3*	*myb3* plants have higher salt-stress tolerance	LF: *PAL1*, *C4H*, *COMT*, *4CL3*, *DFR*, *LDOX, TT8* and EGL3 were up-regulated	(Kim et al., 2022)
	SND1	*snd1* plants have a low tolerance to salt stress	SND1 regulates *Myb46* and *ABI4.*	(Jeong et al., 2018)
*Brassica oleracea*	*CESA1* and *CESA6*	*cesA1* and *cesA6* plants have higher salt-stress tolerance, anatomical and ultrastructural changes in leaves and chloroplasts, dwarf phenotype, reduced content of the cellulose (40%) and pectin (19%), higher soluble sugar content and 3 times higher proline content	LF: *PIP2;2* and *PIP2;3* were 6-7 times up-regulated	(Li et al., 2017)
*Nicotiana tabacum*	*XTH*	Ex plants have a higher rate of root growth under salt-stress conditions and improved tolerance to frost and heat stresses	–	(Kuluev et al., 2017)
*Oryza sativa*	*UGE3*	*uge3* plants have diverse growth defects, hypersensitive to osmotic and salt stresses. *UGE3* Ex plants have increased biosynthesis of cellulose and hemicelluloses, increased tolerance to osmotic and salt stresses, improved Na^+^ and K^+^ homeostasis and higher accumulation of soluble sugars	–	(Tang et al., 2022)
	*CSLD4*	*nd1* plants are salt-stress sensitive but insensitive to ABA treatments. ABA treatment complemented *nd1* salt-stress sensitive phenotype. *CSLD4* OE plants have increased ABA content and enhanced rice salt-stress tolerance	LF: repressed ABA biosynthesis genes OE: up-regulated ABA biosynthesis genes	(Zhao et al., 2022).
	*EIL2*	*EIL2* Ex plants have a low tolerance to salt and drought stresses, delayed leaf development and decreased pectin content. *EIL2* LF plants have enhanced tolerance to salt and drought stresses and delayed leaf senescence	*EIL2* binds and regulates BURP14 and BURP16.	(Jin et al., 2020)
	*TSD2*	*tsd2* plants have increased accumulation of Na^+^ and a lower level of K^+^ in shoot	LF: KAT1, SOS1 and HKT1 were down-regulated	(Fang et al., 2019)
*Panicum virgatum*	*NAC1*	*NAC1* Ex plants have enhanced tolerance to salt stress and higher cellulose content, reduced Na^+^ and increased K^+^ accumulation in roots and shoots. *NAC1* LF plants are salt stress hypersensitive	Ex: antioxidant defence genes and ion-homeostasis-related genes were up-regulated	(Wang et al., 2019b)
*Capsicum annuum*	*XTH3*	*XTH3 Ex* in tomato plants have increased tolerance to drought and salt stresses		(Choi et al., 2011)
*Populus euphratica*	*XTH*	*PeXTH* Ex in tobacco plants have improved water-retaining capacity, and photosynthesis efficiency and increased the number of mesophyll cells		(Han et al., 2013)
*Diospyros kaki*	*XTH*	*XTH* Ex in Arabidopsis and tomato lead to enhanced tolerance to salt and drought stresses, larger and more irregular cells with a higher density of cell walls and intercellular spaces		(Han et al., 2017)
*Nicotiana tabacum*	*XTH*	*XTH* Ex plants have a higher rate of root growth under salt-stress conditions and improved tolerance to frost and heat stresses		(Kuluev et al., 2017)
*Chorispora bungeana*	*PMEI1*	*PMEI1* and *PMEI13* Ex plants have reduced PMEs tissue activity and increased salt tolerance	*CbPMEI1* and *PMEI13* were repressed by ABA and salt stress	(Chen et al., 2018)
*Amaranthus hypochondriacus*	*DGR2*	*AhDGR2* Ex in Arabidopsis causes general abiotic stress intolerance, hypersensitivity to salt stress and ABA treatment		(Palmeros-Suárez et al., 2017)
*Camellia sinensis*	*F3H*	*F3H* Ex in tobacco increased tolerance to salt stress, reduced electrolyte leakage and MDA levels, increased the content and activity of antioxidant enzymes		(Mahajan and Yadav, 2014)
*Betula platyphylla*	*MYB46*	*MYB46 Ex* plants have improved tolerance to salt and osmotic stresses, increased lignin deposition and secondary cell wall thickness; *myb46* plants were hypersensitive to salt and osmotic stresses	Ex: *SOD*, *POD*, *P5CSs* and genes related to the secondary cell wall formation were up-regulated, *P5CDH* and *ProDH* were down-regulated.	(Guo et al., 2017)
	*NAC012*	*BpNAC012* Ex plants have enhanced lignin accumulation; *nac012* plants have reduced secondary cell wall thickening	Ex: *P5CSs*, *SOD POD, and* lignin biosynthesis genes were up-regulated	(Hu et al., 2019)
*Apium graveolens*	*NAC1*	*NAC1* Ex in Arabidopsis plants leads to increased salt and drought stresses tolerance, reduced MDA level	OE: *LAC*, *F5H*, *CCoAOMT*, *C3’H*, *COMT*, *CCR*, SOD and POD were up-regulated	(Duan et al., 2020)
*Fagopyrum tataricum*	NAC16	*NAC16* Ex in Arabidopsis plants leads to salt stress hypersensitivity	Ex: many lignin biosynthesis-related genes were down-regulated; *4CL2* and *CCR2* were up-regulated	(Wang et al., 2021)
*Bryum argenteum*	*DBL1*	*BaDBL1* Ex in Arabidopsis plants leads to increased salt and osmotic stresses tolerance, and the activity of POD, SOD and CAT was increased	Ex: lignin-biosynthesis-related genes and abiotic stress-related TFs and genes (*LEA*, *AtCOR15A* and *AtRD29A*) were up-regulated	(Liang et al., 2021)
*Malus domestica*	*SND1*	*SND1* Ex plants have enhanced resistance to salt and osmotic stresses, and higher lignin and antioxidant content; *snd1* plants were sensitive to salt and osmotic stresses	*SND1* binds *MdMYB46/83, MdRD22*, *MdRD29A*, *MdDREB2A*, *MdAREB1B* and *MdAREB1A*	(Chen et al., 2020)
*Potentilla atrosanguinea*	*SOD*	*SOD* and *APX* Ex in Arabidopsis plants enhanced salt-stress tolerance and increased lignin accumulation, levels of compatible solutes, higher biomass production, better growth rate and increased yield	Double Ex: many lignin biosynthesis-related genes were up-regulated, while *CcAOMT1*, *4CL8*, *CAD1*, *4CL3* and *LAC12* were down-regulated; cell wall-related TFs were up-regulated (VND1, VND2, VND4, VND6, SND1, SND2 and NST1)	(Shafi et al., 2015).

Ex, expression; LF, loss of function.

Cellulose micro-fibrils are β-1,4 linked glucose polymer that is synthesised at the cell surface by cellulose synthase (CesA) complexes (CSCs) not only by higher plants but also algae, some bacteria and even animals, thus, making it the most abundant polymer on the planet (Allen et al., 2021). CesAs are the key part of CSCs, which are active only in the plasma membrane, while actively communicating with the Golgi apparatus and other intracellular compartments *via* exocytosis (trafficking to the plasma membrane) and endocytosis (removal from the plasma membrane). Microtubules and actin microfilaments serve as tracks along which motor proteins transport cell wall components in vesicles. The direct interactions between microtubules, microtubule-linking proteins, and cellulose synthases made the proper microtubules and microfilaments organisation critical for the positioning and construction of cell wall components (Colin et al., 2023). At the same time, the genes in CesA family are specialised, with CesA1, CesA3, and CesA6 participating in primary cell wall synthesis, while CesA4, CesA7, and CesA8 are involved in secondary cell wall synthesis. Also, some other proteins, such as CSI1 (cellulose synthase–interacting protein 1) and CC1-2 (companion of cellulose synthase 1 and 2), are also required for cellulose biosynthesis (Zhu and McFarlane, 2022). Another recently identified members of the CSC are TTL (tetratricopeptide thioredoxin-like) proteins, which interact with CesA1 and cortical microtubules to promote their polymerisation, thus maintaining cellulose synthesis under salt stress conditions (Kesten et al., 2022).

Experimental data from the model plant *Arabidopsis thaliana* [(L.) Heynh.] demonstrated that c*esA1* and c*esA6* single mutants are salt sensitive, with severe root tip swelling, reduced cellulose contents, and root elongation. Mutant lines of *csi1*, which are known to directly interact with CesA6 and cortical microtubules, are also hyper-sensitive to salt stress (Zhang et al., 2016). Similarly, to Arabidopsis, RNAi knockdowns of *CesA1* and *CesA6* in broccoli (*Brassica oleracea* L.) resulted in anatomical and ultrastructural changes in leaves and chloroplasts, dwarf phenotype, and reduced content of the cellulose and pectins (40% and 19%, respectively). However, mutant plants had higher soluble sugar content and three times higher proline content compared with control plants. Furthermore, mutants have higher salt-stress resistance, which was associated with six to seven times up-regulated expression of salt-tolerance–related aquaporins PIP2;2 and PIP2;3 (Li et al., 2017). Thus, these genes might play the role of the negative regulator in plant osmoprotection.

CC1 and 2 proteins could interact with CESAs and microtubules and promote microtubule formation and dynamics. Mutations of the CC1 and 2 resulted in salt-sensitive phenotypes with altered microtubule and CSC behaviour *in vivo.* Under salt, stress CSCs are quickly dissociated from the plasma membrane, and functional CC proteins are necessary to maintain CesA migration and microtubule stability to reassemble CSCs during the growth recovery phase after salt treatment (**Figure 1B**) (Endler et al., 2015). Interestingly, plant CC1 implements an evolutionally conserved microtubule-binding mechanism, similar to that of Tau protein, which is associated with Alzheimer’s disease and known to self-aggregate and trigger neurodegeneration. In particular, two tyrosine residues in the N-terminal region of CC1 are responsible for the microtubule binding, both *in vitro* and *in vivo*. Point mutations in these residues interrupted normal microtubule-guided CSC movement and resulted in the generation of a salt stress-hyper-sensitive microtubule array (Kesten et al., 2019).

Recently, an ubiquitously expressed and salt-stress induced gene *UGE3* (UDP-galactose/glucose epimerase 3) from rice, which provides substrates for polysaccharides polymerization, was demonstrated to improve biomass production, mechanical properties of the cell wall, and increased tolerance to salt and osmotic stresses. *uge3* mutants displayed diverse growth defects and were hypersensitive to osmotic and salt stresses. On the other side, *OsUGE3* overexpressing plants had increased biosynthesis of cellulose and hemicelluloses, which resulted in improved mechanical strength of the cell wall. Furthermore, *OsUGE3* overexpressors showed increased tolerance to osmotic and salt stresses, which was associated with improved Na^+^ and K^+^ homeostasis and higher accumulation of soluble sugars (Tang et al., 2022).

Additionally, some other cellulose biosynthesis-related proteins have also been reported involved in salt tolerance. For example, KORRIGAN1 (KOR1), a membrane-anchored endo-β-1,4-glucanase, is an integral part of the primary cell wall, physically interacting with CSC and linking cell wall biosynthesis and abiotic stress tolerance. KOR1 was shown to cycle between the trans-Golgi network and the plasma membrane under normal conditions. While under salt stress, this cycling is interrupted, and KOR1 is retained in the plasma membrane or transported to the tonoplast for degradation, thus unable to support cellulose biosynthesis. Not surprisingly, *kor1* mutants have the salt-stress sensitive phenotype, cellulose deficiency, and retarded growth (Nagashima et al., 2020).

**Figure 3:**
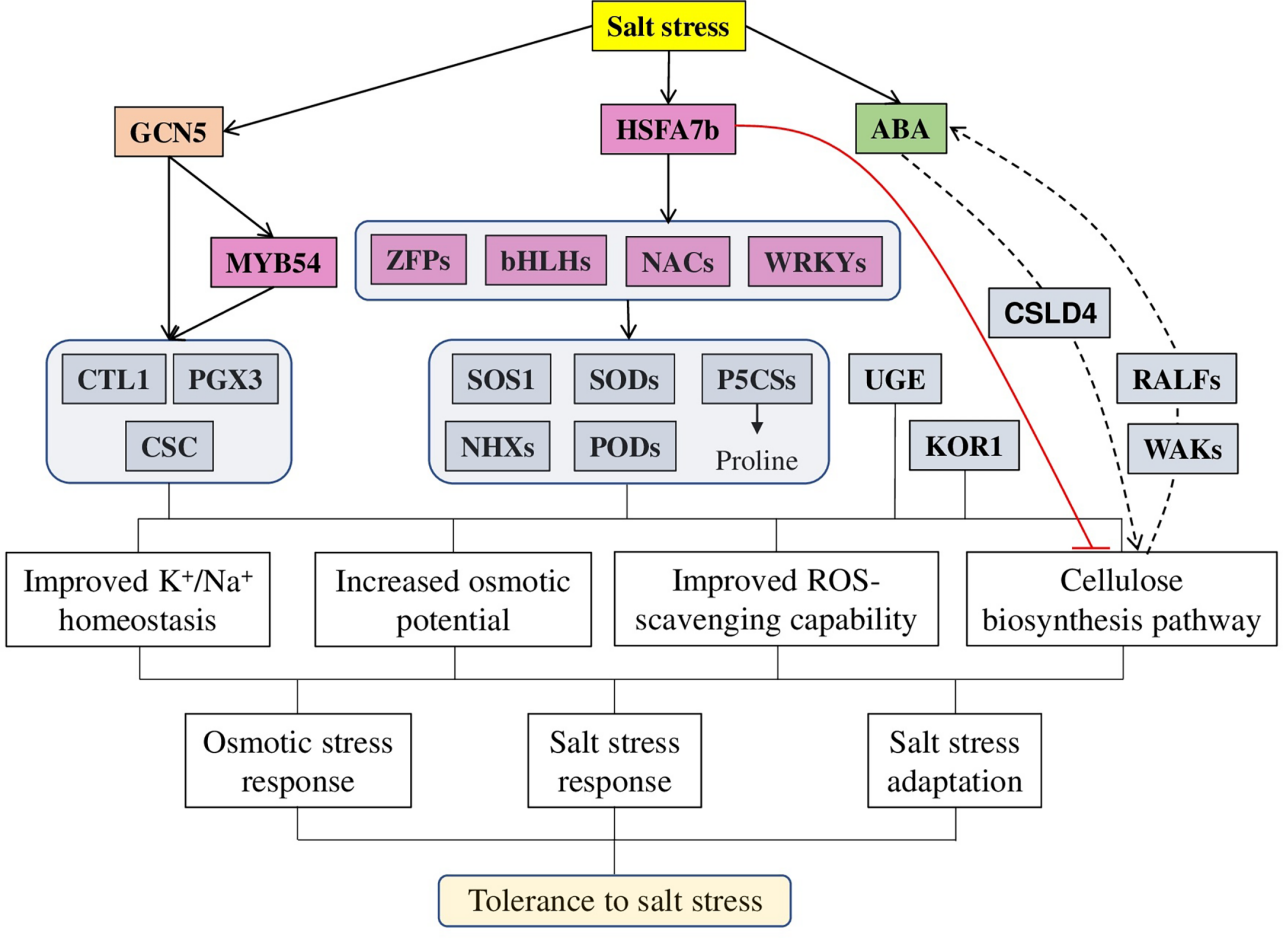
A model of different factors regulating cellulose-associated salt stress tolerance in plants. Salt stress induces the expression of a master regulator HSFA7b (heat shock transcription factors), which activates a wide range of genes, such as other TF (from WRKY, NAC, HLH, and ZFP families), ion transporters (SOSs and NHXs), antioxidant defence (SODs, GSTs, and PODs) and osmoprotectors (P5CSs) while inhibiting cellulose synthesis related genes (CesAs, CSLAs, and CSLGs). Similarly, histone acetyltransferase GCN5 (general control non-repressed protein 5) regulates adaptation to salt stress *via* directly targeting *MYB54* and cellulose biosynthesis–related genes [*CTL1* (chitinase-like gene) and *PGX3* (polygalacturonase involved in expansion 3)]. Additionally to other genes, directly affecting the cellulose biosynthesis process [such as *KOR1* (*KORRIGAN1*) and *UGE3* (UDP-galactose/glucose epimerase 3], genes like *CSLD4* modulate the activity and location of cell wall-localised proteins (WAKs and RALFs) to sustain high ABA content to enhance salt and osmotic stress tolerance. In total, all these target genes provide various physiological changes, leading to improved salt stress tolerance: improving ions homeostasis and ROS scavenging capability, increasing osmotic potential and modulating cellulose biosynthesis. The red line represents negative regulation; the dashed line represents other involved components.

Recently, several mechanisms regulating cellulose biosynthesis in relation to salt-stress tolerance have been identified (**Figure 3**). For example, overexpression of the NAC1 TF (transcription factor) in Switchgrass (*Panicum virgatum* L.) leads to higher cellulose content, enhanced tolerance to salt stress, and up-regulated expression of antioxidant defence and ion-homeostasis–related genes, reduced accumulation of Na^+^ and increased of K^+^ in roots and shoots. At the same time, *NAC1* RNAi plants were hyper-sensitive to salt stress (Wang et al., 2019b). Arabidopsis AtHSFA7b [heat shock factor (class A)–type transcription factor], known to participate in abiotic stress responses, is also involved in salt tolerance. Under salt conditions, AtHSFA7b improved salt tolerance by regulating genes, such as *SOS1*, *NHXs*, *PODs* (peroxidase), and *SODs* (superoxide dismutase). These gene interactions lead to the reduction of water loss rate, adjustment of osmotic potential, and a decrease in the accumulation of reactive oxygen species. In particular, the content of proline and soluble sugars was increased after NaCl treatment in an AtHSFA7b-dependent way. Under salt-stress conditions, plant lines overexpressing *AtHSFA7b* had increased levels of proline biosynthesis genes (*AtP5CS1* and *AtP5CS2*, encoding Δ1-pyrroline-5-carboxylate synthases), while *hstfa7b* plants had lower *AtP5CS1* and *AtP5CS2* expression. Several other TFs, positive regulators of salt stress, were regulated by AtHSFA7b: bHLH, NAC061, NAC036, NAC090, WRKY38, and ZFP2 (Zinc finger protein). Finally, 31 cellulose biosynthesis-related genes [including *CESA8*, *CSLG1* (cellulose synthase-like glycosyltransferase), *CSLG2*, and *CSLA9*] were down-regulated by AtHSFA7b, suggesting these genes as negative modulators of salt tolerance (Zang et al., 2019).

Epigenetic mechanisms also play an important role in salt tolerance. As it was recently demonstrated, the mRNA levels of histone acetyltransferase GCN5 (general control non-repressed protein 5) were increased after salt treatment, thus suggesting its importance in maintaining the cell wall integrity under salt stress. Several genes, such as *CTL1* (chitinase-like gene), *PGX3* (polygalacturonase involved in expansion 3), and *MYB54* (MYB domain protein 54), involved in cellulose biosynthesis, were identified as direct GCN5 targets. Confirming these results, the *gcn5* mutants exhibited reduced cellulose content, severe growth inhibition, and cell wall anomaly under salt stress. Furthermore, the salt tolerance and cell wall integrity phenotypes of the Arabidopsis *gcn5* mutant were complemented by the expression of the wheat *TaGCN5* gene, suggesting a conserved role of the GCN5-mediated salt tolerance between these two species (Zheng et al., 2019).

Additionally, the plant hormone ABA, known to regulate various aspects of plant growth, development, and stress responses, is also linked to cellulose synthesis-regulated salt stress tolerance. The rice OsCSLD4 (cellulose synthase-like D4 protein) mutant (*nd1*) was sensitive to salt stress but insensitive to ABA treatments. Interestingly, the expression of some ABA synthesis and signaling genes was repressed in *nd1* mutant under both normal and salt stress conditions. Exogenous ABA treatment effectively complemented *nd1* salt-stress sensitive phenotype. Furthermore, *OsCSLD4* overexpression leads to the up-regulated expression of ABA biosynthesis genes, increased ABA content and enhanced rice salt-stress tolerance, suggesting that the role of OsCSLD4 in salt stress tolerance is ABA-mediated (Zhao et al., 2022).

### 2.2 Hemicelluloses

Hemicelluloses are branched heteropolymer interacting and cross-linking cellulose microfibrils, thus strengthening plant cell walls (Khodayari et al., 2021). A recent study on vascular plants suggested that hemicelluloses could also interact with lignin in some cell walls (Gallina et al., 2018). The abundance and structure of hemicelluloses vary greatly in different plant species, with main examples grouped into xylan, xyloglucan, arabinoxylan, glucuronoxylan, and glucomannan (Scheller and Ulvskov, 2010). Xyloglucan is involved in cell wall strengthening *via* cellulose microfibrils binding during cell elongation, while xylan participates in polysaccharide cross-linking during cell wall architecture establishment. Modification of hemicelluloses by the cell wall remodelling enzymes is an important way to control cell wall extensibility, and XTHs (xyloglucan endotransglucosylase/hydrolases) is the best-studied family facilitating cell wall plasticity (Ishida and Yokoyama, 2022). XTHs can cleave and/or reconnect xyloglucans, which leads to the attachment of the reducing end of the xyloglucans to the non-reducing end of another xyloglucans, thus producing chimeric oligomer or polymer xyloglucan molecules (Park and Cosgrove, 2015). XTHs homologues react to salt stress differently, with some homologues up-regulated, while other – down-regulated and some did not change their level of expression. Therefore, further identification and detailed investigation of every XTHs’ (both positive and negative) regulator would help to create new plants with improved tolerance to salt stress.

Early research demonstrated that XTHs are active players in various abiotic stresses, including high-salinity stress. Experiments on *Medicago truncatula* demonstrated that XTHs responded differently to salt stress, with eight up-regulated and 11 down-regulated genes (Xuan et al., 2016). Similar results were reported also for poplar (*Populus* sp.), where 11 differently expressed genes were identified in the roots (five up- and six down-regulated), nine in the stems (four up- and five down-regulated), and seven in the leaves (four up- and three down-regulated) after salt treatment (Cheng et al., 2021). These data were confirmed on other species such as typical halophyte, *Salicornia europaea*, where salt stress up-regulated expression of 27 and 15 SeXTHs (in shoots and roots, respectively) (Tiika et al., 2021), and *Vitis vinifera* L., four VvXTHs were observed (Qiao et al., 2022). Therefore, XTHs’ expression is tissue specific, and some members of the family are positive regulators of salinity tolerance, while others are negative.

Thereby, transgenic tomato plants expressing pepper (*Capsicum annuum* L.) *CaXTH3* demonstrated an increased tolerance for drought and salt stresses (**Table 1**). Transgenic plants had normal phenotype under standard growth conditions and also no significant difference in chlorophyll content and root elongation under salt stress (Choi et al., 2011). Similarly, the expression of poplar (*Populus euphratica* Oliv.) *PeXTH* in tobacco improved water-retaining capacity and photosynthesis efficiency and increased the number of mesophyll cells under salt-stress conditions (Han et al., 2013). Transgenic Arabidopsis and tomato plants expressing persimmon (*Diospyros kaki* Thunb.) *XTH* had larger and more irregular cells with a higher density of cell walls and intercellular spaces, which resulted in enhanced tolerance for salt and drought stresses (Han et al., 2017).

A negative role in salt tolerance was reported for Arabidopsis *XTH30*, which was strongly up-regulated under salt stress in the stem, root, flower, and hypocotyl. However, plants overexpressing *XTH30* were hyper-sensitive to salt stress, while loss-of-function *XTH30* mutant demonstrated lower Na^+^ accumulation in shoot and H_2_O_2_ content and increased salt tolerance (Yan et al., 2019).

In addition to diverse abiotic stresses, the expression of *XTH* is regulated by plant hormones. For example, *XTH* from tobacco (*Nicotiana tabacum* L.) was up-regulated by both ABA treatment (10 μM) and different stresses (drought, salt, and cadmium), while down-regulated by higher ABA concentration (100 μM) and 0^°^C stress, thus suggesting its implication in ABA-dependent stress signaling. At the same time, *NtXTH* overexpressing tobacco plants demonstrated a higher rate of root growth under salt-stress conditions, and also greater frost and heat tolerance when compared with control plants (Kuluev et al., 2017). In Arabidopsis, *XTH19* and *XTH23* were induced by salt stress *via* the brassinosteroid signaling TF BES1 (BRI1-EMS-SUPPRESSOR 1). *xth23* single and *xth19/xth23* double mutant were additively sensitive to salt stress, while *BES1* overexpression increased salt tolerance in wild-type and partially complement salt tolerance and phenotype of *xth19/xth23* mutant. Further experiments showed that *XTH19* and *XTH23* expression is directly regulated by BES1, thus postulating a novel role for brassinosteroids in regulating XTH-mediated salt sensitivity (Xu et al., 2020).

### 2.3 Pectins

Pectins are acidic polysaccharides enriched with α-(1,4)–linked galacturonic acids and accounted for up to 40% of the higher plant cell wall content. Pectins play a crucial role in plant growth and development, immunity, response to stresses, and senescence [reviewed in (Cruz-Valderrama et al., 2019; De Lorenzo et al., 2019; Dauphin et al., 2022)]. The three major types of pectins are HG (homogalacturonan), RG-I (rhamnogalacturonan-I), and RG-II (rhamnogalacturonan-II), which are selectively modified by PMEs (pectin methyl esterases), PAEs (pectin acetylesterases), PGs (polygalacturonases), or PLLs (pectate lyases–like) during cell growth or in response to stresses. The dynamic regulation and interplay between expression and activity of modified enzymes and their inhibitors [such as PMEIs (PME inhibitors) and EGCG (epigallocatechin gallate)] define the extent of cell walls stiffness [reviewed in (Coculo and Lionetti, 2022)] (Du et al., 2022). Different pectin-modified enzymes have been shown to act as positive or negative regulators of salt tolerance in plants.

Thereby, in tobacco (*Nicotiana tabacum* L.) *NtPME005*, *NtPME039*, *NtPME043*, *NtPME047*, *NtPME082*, *NtPME106*, and *NtPME108* were induced and up-regulated, while *NtPME029*, *NtPME056*, *NtPME058*, and *NtPME062* were down-regulated by salt stress. Interestingly, some *NtPME* genes (*NtPME005*, *NtPME039*, and *NtPME106*) were down-regulated by ABA treatments and up-regulated by NaCl stress, suggesting the involvement of ABA pathway in salt stress tolerance (Sun et al., 2022). Expression *PMEI1* from *Chorispora bungeana* (Fisch.) and its Arabidopsis homologue *PMEI13* in Arabidopsis reduced PMEs tissue activity and increased salt tolerance. Interestingly, both *CbPMEI1* and *PMEI13* genes were repressed by salt stress and ABA treatments, thus further supporting the involvement of ABA in PME-mediated salt tolerance regulation (Chen et al., 2018).

Recently, another ABA-mediated mechanism of salt-stress tolerance was demonstrated on rice (*Oryza sativa* L.). In particular, OsEIL2 (EIN3-Like) (ETHYLENE-INSENSITIVE 3), a nuclear-localised TF induced by salt stress and ABA treatment, directly binds and regulates genes encoding β subunit of polygalacturonase subfamilies (OsBURP14 and OsBURP16) (**Table 1**). *OsEIL2* overexpression resulted in delayed leaf development, decreased (because of the increased PG activity) pectins content and reduced tolerance to salt and drought stresses. On the contrary, *OsEIL2* RNAi knockdown mutant demonstrated delayed leaf senescence and enhanced tolerance to salt and drought stresses (Jin et al., 2020). Another conserved gene, *DGR2* (DUF642 L-GALACTONO-1,4-LACTONE-RESPONSIVE) from *Amaranthus hypochondriacus* (L.), encoding DUF642 domain–containing protein induced by drought and salt stresses, was also associated with cell wall modifications. *AhDGR2* overexpression in Arabidopsis led to hypersensitivity to NaCl and ABA, and general abiotic stress intolerance. Interestingly, the effect of *AhDGR2* overexpression on the PMEs activity was organ-specific, significantly lower in leaves but higher in roots, thus suggesting further insights into the precise regulation of cell wall modifications (Palmeros-Suárez et al., 2017). Similarly, overexpression of tea [*Camellia sinensis* (L.) Kuntze] *CsF3H* gene, encoding flavanone 3-hydroxylase in tobacco, increased tolerance to salt stress *via* decreased PMEs activity in roots and leaves. Also, transgenic plants demonstrated reduced electrolyte leakage and MDA (malondialdehyde) levels, while increasing the content and activity of antioxidant enzymes (Mahajan and Yadav, 2014).

PMEs are novel and often overlooked players in the development of salt stress tolerance. PMEs are closely associated with ABA signaling pathways, a wide range of cell-surface sensors and other abiotic stresses-related genes. However, the exact regulatory circuit and molecular mechanism of their action are unknown and require further investigation.

Arabidopsis *PME31* was significantly up-regulated after salt treatment. It was shown that PME31 acts as a positive regulator of salt stress tolerance. Accordingly, *PME31* knockdown resulted in hypersensitivity to salt stress and down-regulation of other genes related to drought and temperature stresses [such as *DREB2A* (dehydration-responsive element-binding protein), *RD29A* (low-temperature–induced 78 kDa protein) and *RD29B*] (Yan et al., 2018). Similarly, the mutation in rice *PME* (OsTSD2) reduced the expression of several genes maintaining ion homeostasis (*OsKAT1*, *OsSOS1*, and *OsHKT1*), which resulted in increased accumulation of Na^+^ and a lower level of K^+^ in shoot under salt stress (Fang et al., 2019).

Additionally, cell walls perceive various stresses with cell-surface localised receptors, such as WAKs (wall-associated kinases), LyKs (LysM receptor-like kinases), LRR-RLKs (leucine-rich repeat receptor-like kinases), LRXs (Leucine-rich repeat extensins) and CrRLK1L (*Catharanthus roseus* receptor-like kinase 1-like) [reviewed in (Bacete et al., 2018)]. FERONIA (FER) is a plasma membrane–localised receptor kinase perceiving salt-stress–mediated damage to the pectins-associated wall. Therefore, root cells of *fer* mutant were shown to explode during growth recovery after salt treatment. Conducted *in vitro* experiments proposed a mechanism where FER directly interacts with pectins, senses salinity-mediated softening of the cell wall and initiates a Ca^2+^-mediated signaling cascade preventing root cells from exploding during growth under salt stress. The exposure to the salt stress causes the surplus of Na^+^ to replace Ca^2+^ in pectins binding, thus interrupting normal cross-linking of pectins and reducing cell elongation, Ca^2+^ or borate treatment could rescue *fer*-associated phenotype and facilitating pectins cross-linking (Feng et al., 2018). Later studies provide evidence of the close association between FER and LRX3/4/5 (Dünser et al., 2019), and RALF22/23 (RAPID ALKALINIZATION FACTOR) (Yu and Assmann, 2018), thus proposing their functioning as a LRX-RALF-FER module to transduce cell wall signals plant growth in normal condition and tolerance to salt stress (**Figure 1B**) (Zhao et al., 2018). Furthermore, recent research has identified that LRX-RALF-FER module regulates the homeostasis of jasmonic acid, salicylic acid and abscisic acid, thus controlling plant salt stress response (Zhao et al., 2021). Later, a similar mechanism was described for other sensors, such as THE1 (THESEUS1) and HERK1 (HERKULES1) (Gigli-Bisceglia et al., 2022), WAK2 [*Brachypodium distachyon* (L.) P.Beauv.], and AtWAK2 (Wu et al., 2020). In particular, while *the1* and *herk1* single mutants have wild-type salt stress phenotype, the double mutants have hypersensitive to salt stress phenotype, similar to *fer* mutants. In the proposed model THE-HERK sensors mitigated salt stress through the negative regulation of MAPK6 (mitogen-activated protein kinase 6) (Gigli-Bisceglia et al., 2022), which is known to regulate many cellular processes, including also salt stress response (Zhang and Zhang, 2022).

### 2.4 Lignin

**Figure 4:**
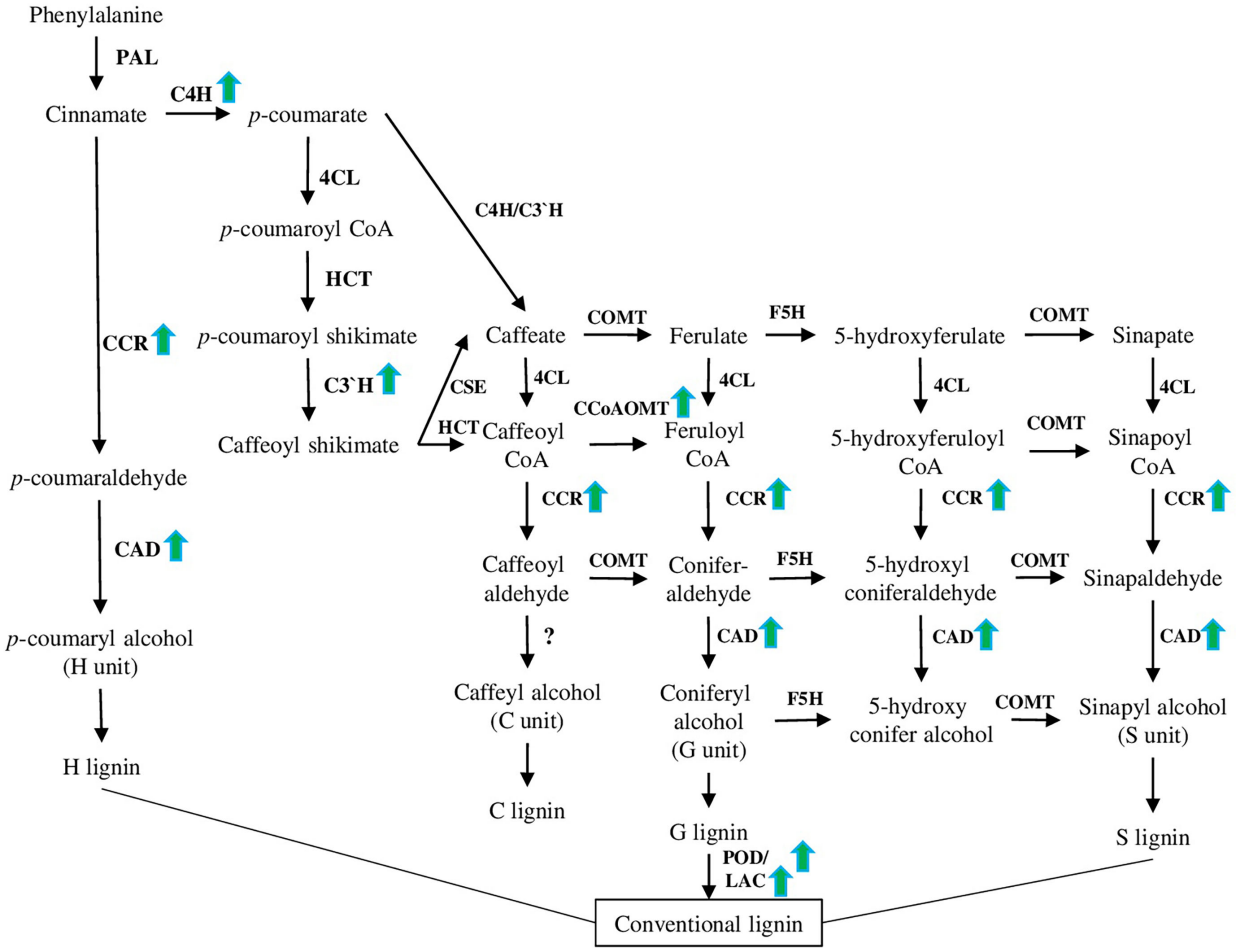
Lignin biosynthesis pathway. This pathway has been modified based on recently published papers (please see the main text of section 4.4). PAL (phenylalanine ammonia-lyase), C4H (cinnamate 4-hydroxylase), 4CL (4-coumarate: CoA ligase), CCR (cinnamoyl CoA reductase), CCoAOMT (caffeoyl CoA O-methyltransferase), F5H (ferulate 5-hydroxylase), COMT (caffeate 3-O-methyltransferase), and CAD are involved in the synthesis of monolignols. POD (peroxidase) and LAC (laccase) are involved in the polymerization of monolignols to yield the lignin polymer as a final product. Green arrows represent genes up-regulated in different species under salt stress conditions according to recent papers [(Li et al., 2021), (Isayenkov et al., 2020), (Chun et al., 2019), and (Vasupalli et al., 2021)].

Lignin is the main structural component of the plant cell wall, composed of the units derived from the polymerisation of sinapyl, coniferyl, *p*-coumaryl, and hydroxycinnamyl alcohols (**Figure 4**). Accumulation of lignin and cell wall thickening because of the activation of the lignin biosynthesis pathway is an important response to various environmental stresses, including high salinity [reviewed in (Vanholme et al., 2019; Coomey et al., 2020)].

#### 2.4.1 Transcriptional regulation of lignin biosynthesis

Salt-mediated activation of lignin biosynthesis and deposition processes are tightly regulated and associated with other mechanisms of abiotic stress tolerance: antioxidant defence, anthocyanin and ABA biosynthesis. At the same time, some TFs play a more important role than others, activating lignin biosynthesis and a wide range of other salt stress-specific and general abiotic stress response mechanisms. Such master regulators TFs (SND1 or NAC83) have great potential as targets for the development of salt-stress tolerant plant species.

**Figure 5:**
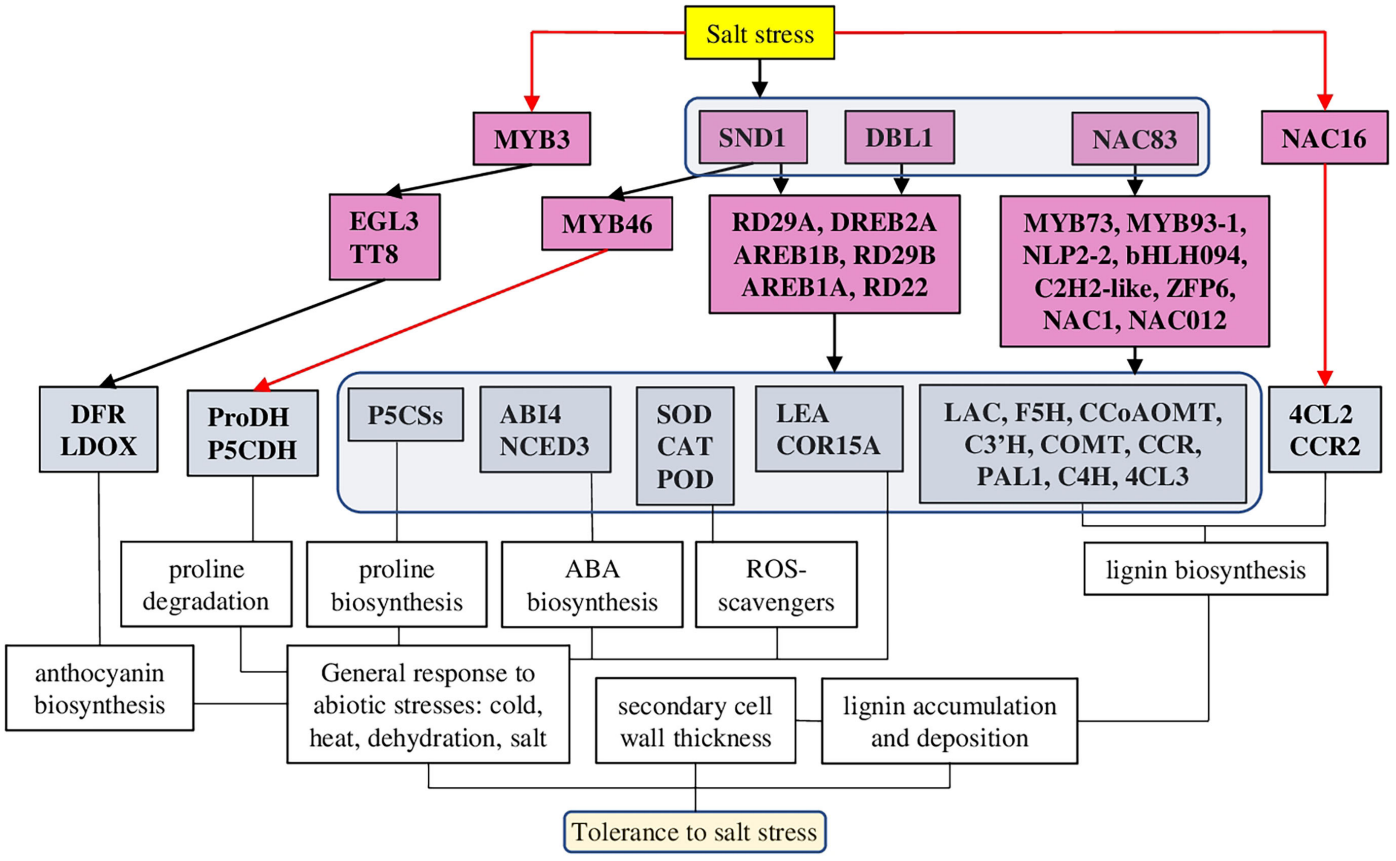
A model of different TFs regulating lignin-associated salt stress tolerance in plants. TFs of several families have been identified as salt-mediated activators of lignin biosynthesis and deposition. Among them, several TFs (SND1, DBL1, and NAC83) have been defined as master regulators, which, in addition to the lignin biosynthesis pathway, were activating also other TFs associated with general stress responses, antioxidant defence, ABA, and anthocyanin biosynthesis. The red line represents negative regulation.

A number of transcriptomic studies on different species suggested that up-regulation of the lignin biosynthesis pathway and antioxidant defence are common responses to salt stress (**Figure 5**). In particular, 28 and 23 differentially expressed genes related to lignin and flavonoid biosynthesis pathways, respectively, have been identified in *Sophora alopecuroides* (L.) after salt treatment (Zhu et al., 2021). Among other cell wall metabolism-related genes, 10-fold up-regulation of laccases was shown on halophytic barley *Hordeum marinum* ssp. *marinum* under salt stress conditions (Isayenkov et al., 2020). Also, many lignin biosynthesis-related genes (such as POD, CAD (cinnamyl alcohol dehydrogenase), C4H (cinnamate 4-hydroxylase), CcoAOMT (caffeoyl CoA O-methyltransferase), CCR (cinnamoyl CoA reductase) and C3′H (*p*-coumarate 3-hydroxylase) (**Figure 4**), and 36 TFs associated with various families (such as HSF, MYB, NAC, WRKY, bZIP, AP2/ERF-ERF, and C2H2) have been identified in halophytic plant *Eutrema salsugineum* (Pall.) (Li et al., 2021). Moreover, transcriptome profiling of two garlic (*Allium sativum* L.) cultivars (salt sensitive and salt tolerant) revealed that under salt stress, most transcripts of the phenylpropanoid biosynthesis pathway were down-regulated in the salt-sensitive genotype. Additionally, many transcripts related to the brassinosteroid signaling and biosynthesis pathways were down-regulated in the salt-sensitive cultivar (Kong et al., 2021). Interestingly, in a similar experimental setup, genes related to other hormones (such as auxin, ethylene, brassinosteroid, abscisic acid, and jasmonate) demonstrated different time-dependent expression patterns (such as auxin-induced protein 22D, auxin response factor, ethylene-responsive transcription factor, ethylene receptor 2–like, protein phosphatase 2C, BES1/BZR1, BRI1, and ZIM domain–containing protein) (Wang et al., 2019a). Furthermore, we discuss recent results dedicated to the defined signaling pathways regulating lignin accumulation to strengthen cell walls and protect membrane integrity under salt stress.

Genome-wide examination of the R2R3-MYB TF family in pigeon pea [*Cajanus cajan*, (L.) Millsp.] identified 30 R2R3-MYB TFs activated by abiotic stresses and involved in lignin and flavonoid biosynthesis. Interestingly, another 122 key enzyme genes from flavonoid and lignin biosynthetic pathways were MeJA (Methyl jasmonate) responsive (Yang et al., 2021).

BpMYB46, the member of the plant MYB transcription family, was characterised in *Betula platyphylla* (Sukaczev.) as associated with abiotic stress tolerance and secondary cell wall biosynthesis (**Table 1**). Therefore, *BplMYB46* overexpression in plants resulted in increased expression of ROS scavengers (*SOD* and *POD*) and proline biosynthesis genes (*P5CSs*), while proline degrading genes (*P5CDH* and *ProDH*) were down-regulated. Also, *BpMYB46* overexpressing lines showed increased lignin deposition, secondary cell wall thickness, up-regulated genes related to the secondary cell wall formation and reduced stomatal apertures and water loss. In total, mutant lines had improved tolerance to salt and osmotic stresses, while *BpMYB46* knockdown lines were hyper-sensitive to these stresses (Guo et al., 2017).

Interestingly, Arabidopsis MYB3 TF was identified as a salt-stress–mediated repressor for regulating lignin and anthocyanin biosynthesis. While the expression of many salt response genes was not altered in *myb3* plants, the expression of anthocyanin and lignin biosynthesis genes (such as *PAL1* (phenylalanine ammonia-lyase 1), *C4H*, *COMT* (caffeic acid O-methyltransferase), *4CL3* (4-coumarate–CoA ligase 3), *DFR* (dihydroflavonol 4-reductase), and *LDOX* [leucoanthocyanidin dioxygenase)] and anthocyanin regulators TFs [such as TT8 (Transparent Testa 8) and EGL3 (Enhancer of Glabra 3)] was increased (Kim et al., 2022).

Several members of the NAC transcription family have been characterised as regulators of the expression of lignin-related and salt stress-responsive genes. Indeed, network reconstruction of the lignocellulosic pathway in *Populus davidiana* × *Populus bolleana* under salt stress revealed the involvement of 197 diverse TFs. However, PdbNAC83 was identified as the up-top TF regulating other salt stress- and lignocellulosic synthesis–related genes, including also TFs (*PdbMYB73*, *PdbMYB93-1*, *PdbNLP2-2*, *PdbC2H2-like*, *PdbbHLH094*, and *PdbZFP6*) (Lei et al., 2022).

Analysis of overexpressing and knockdown lines of *NAC012* suggested its function associated with osmotic and salt stress tolerance. Similarly to BpMYB46, BpNAC012 induced the expression of *P5CSs*, *SOD*, and *POD* predominantly in mature stems. Under salt and osmotic stresses, *BpNAC012* overexpressing lines had higher expression levels of lignin biosynthesis genes and enhanced lignin accumulation. On the contrary, knockdown lines greatly reduced the secondary cell wall thickening of stem fibres (Hu et al., 2019). Overexpression of *AgNAC1* (*Apium graveolens* L.) in Arabidopsis resulted in increased tolerance to salt and drought stresses through up-regulation of lignin biosynthesis–related genes [such as *AtLAC* (laccase), *AtF5H* (Ferulate-5-hydroxylase), *AtCCoAOMT*, *AtC3’H*, *AtCOMT*, and *AtCCR*], antioxidants (SOD and POD), and reduced level of MDA (Duan et al., 2020).

Finally, in Arabidopsis, the key NAC family TF SND1 (SECONDARY WALL-ASSOCIATED NAC DOMAIN PROTEIN 1) was characterised as the master regulator, connecting abiotic stress tolerance and secondary cell wall biosynthesis through regulation of abscisic acid levels and activation of other transcription factors. In particular, SND1 was shown to bind *Myb46*, thus activating the lignin biosynthesis pathway, and to directly bind the *ABI4* gene, thus reducing ABA levels. Also, *snd1* knockdown mutants exhibited a low tolerance to salt stress (Jeong et al., 2018). Furthermore, experiments on apple (*Malus* × domestica Borkh.) suggested that *MdSND1* was activated by both ABA and salt stress and bounded directly not only to *MdMYB46/83* but also other stress-related TFs such as *MdRD22*, *MdRD29A*, *MdDREB2A*, *MdAREB1B*, and *MdAREB1A*, thus activating response to a wide range of stresses. Indeed, *MdSND1* overexpressing plants had higher lignin and antioxidant content and enhanced resistance to salt and osmotic stresses, while *MdSND1* knockdown plants were stress sensitive (Chen et al., 2020).

On the contrary, FtNAC16 [*Fagopyrum tataricum* (L.) Gaertn.] was identified as a negative regulator of salt stress tolerance and lignin biosynthesis. *FtNAC16-*expressing Arabidopsis plants demonstrated reduced expression of many lignin biosynthesis–related genes. At the same time*, 4CL2* (4-coumarate–CoA ligase 2) and *CCR2* genes were abnormally up-regulated, suggesting a functional compensatory mechanism changing the proportions of the lignin monomer, resulting in hypersensitivity to salt stress (Wang et al., 2021).

DREB family TF isolated from desiccation-tolerant moss *Bryum argenteum* (Hedw.) plays important roles in tolerance to abiotic stresses. *BaDBL1-*expressing Arabidopsis plants demonstrated increased tolerance to salt and osmotic stresses, which was associated with increased activities of antioxidant enzymes [POD, SOD, and CAT (Catalase)] and transcription of lignin-biosynthesis–related genes. Additionally, BaDBL1 up-regulated other abiotic stress-related TFs and genes [such as *AtLEA* (late embryogenesis abundant protein), *AtCOR15A* (Protein COLD-REGULATED) and *AtRD29A*) (Liang et al., 2021).

#### 2.4.2 Lignin biosynthesis genes associated with plant salt tolerance

Activation of lignin biosynthesis and deposition pathways plays an important role in the development of salt stress tolerance. However, limited data available suggested that salt stress affects the biosynthesis of lignin units in a different way, leading to shifts mostly between S and G units. Additionally, the antioxidant defence system, abscisic acid, proline, and anthocyanin biosynthesis are greatly contributing to the development of salt stress tolerance.

Analysis of salt-stress up-regulated genes suggested that *CcoAOMT* (caffeoyl CoA O-methyltransferase) is one of the most highly induced genes (**Table 1**). The product of CcoAOMT further leads to the guaiacyl and sinapyl lignin formation (G and S units). Interestingly, *ccoaomt1* plants were hypersensitive to salt stress and had lower lignin content, but the amount of only G monomer was reduced, while the amounts of S and H were higher (**Figure 4**) (Do et al., 2007; Chun et al., 2019). COMT, another crucial enzyme involved in lignin synthesis of S and G monomers, was recently characterised in rice (*Oryza sativa* L.) in relation to salt stress and lignin content. Among 33 identified genes, 5 and 4 *OsCOMTs* were up- and down-regulated under salt stress, respectively. A combined investigation of lignin content and expression analysis of *COMT* genes suggested that *OsCOMT8*, *OsCOMT9*, and *OsCOMT15* as the key players in the synthesis of lignin (Liang et al., 2022).

CAD family is essential for lignin biosynthesis, because it catalyses the final step of all units (H, G, and S) production. As was shown on *Phyllostachys edulis* [(Carrière) J.Houz.], the *PheCAD* family highly co-expressed with biotic and abiotic stress responses, with *PheCAD2*, *PheCAD3*, and *PheCAD5* being the main up-regulated genes after drought and salt stress treatments (**Figure 4**). Among other *PheCAD* genes highly expressed in many tissues (*PheCAD1*, *PheCAD2*, *PheCAD6*, *PheCAD8*, and *PheCAD12*), *PheCAD2* demonstrated a positive correlation with most of the lignin biosynthesis enzymes, suggesting that PheCAD2 might be the main enzyme responsible for lignin biosynthesis (Vasupalli et al., 2021).

ROS scavengers also play a crucial role in salt-stress tolerance and are closely connected to lignin biosynthesis. Among other ROS, H_2_O_2_ is known as a crucial trigger, initiating peroxidase-dependent oxidation of cinnamyl alcohol to lignins (Wen et al., 2020). As it was recently shown, transgenic Arabidopsis overexpressing *SOD* (from *Potentilla atrosanguinea* George Loddiges) and *APX* (L-ascorbate peroxidase) (from *Rheum australe* D.Don) (both individually and combined), had increased lignin accumulation with altered S:G ration and higher levels of compatible solutes, which provided enhanced salt-stress tolerance. In particular, under salt stress conditions, all transgenic plants had twofold higher lignin deposition with a higher S:G ratio. Furthermore, dual transformants had up-regulated expression of many lignin biosynthesis genes under salt stress, while five genes (*CcAOMT1*, *4CL8*, *CAD1*, *4CL3*, and *LAC12)* were down-regulated. Additionally, many cell wall–related TFs were up-regulated [such as VND1 (VASCULAR-RELATED NAC-DOMAIN), VND2, VND4, VND6, SND1, SND2, and NST1 (NAC SECONDARY WALL THICKENING)]. Finally, transgenic plants demonstrated higher biomass production, better growth rate, and increased yield under salt stress when compared to WT plants (Shafi et al., 2015).

## 3 Root barriers of salt transport – Casparian strips and suberinisation of cell walls

### 3.1 Regulation of cell wall suberinisation on the transcriptional level

The walls of specialised cells could be modified in response to salt stress, for example, the suberinisation of the endodermis and exodermis (hypodermis) (Shukla and Barberon, 2021). Such modifications affect the transfer of solutes and water, thus preventing the entrance of surplus Na^+^ and Cl^-^ into the plant and the leakage of K^+^ and water out (Byrt et al., 2018). TFs members of the MYB and WRKY families are the main regulators of suberin biosynthesis and deposition. Suberin metabolism is closely associated with other fatty acid-based pathways, such as cutin and wax biosynthesis.

Recently, MYB family TFs (MYB41, MYB53, MYB92, and MYB93) have been identified as the main regulators of suberin biosynthesis and deposition, each of which is sufficient to endodermal suberin formation in response to both environmental and developmental signals. Indeed, the quadruple mutant of these four TFs had greatly reduced levels of suberin and affected endodermal deposition of lignin (in CS), probably through a compensatory mechanism. Furthermore, mutant plants had increased sensitivity to salt stress and suberinisation, which was not regulated by ABA and salt treatments. Thus, supporting the central role of suberin in plant adaptation to salt stress (Shukla et al., 2021).

Similarly, MYB family TFs regulate suberin assembly also in the seed coat. As it was recently shown, seeds of *myb9* and *myb107* mutants demonstrated lower germination rates and increased permeability under salt stress due to the reduced amount of suberin monomers and changed levels of other seed coat-associated metabolites (Lashbrooke et al., 2016). Also, MYB49 was shown to contribute to salt-enhanced tolerance in Arabidopsis through the transcriptional activation of cutin deposition and antioxidant defence (Zhang et al., 2020). The plant cuticle, composed of cutin and associated waxes, is usually deposited on the surface of the epidermal cell wall of plant leaves and stems (Kosma et al., 2014). Cutin differs from suberin by lower phenolic content, almost exclusively C16 and C18 monomers, and around 50% of C18:2 monomers (Yang et al., 2017). Therefore, *MYB49* overexpressing plants demonstrated significant up-regulation of the ‘cutin, suberin and wax biosyntheses’ category of genes under normal and/or salt stress conditions, while in *myb49* mutants these genes were down-regulated. Several biosynthetic genes, such as *ASFT* (omega-hydroxypalmitate O-feruloyl transferase), *FACT* (fatty alcohol:caffeoyl-CoA acyltransferase), *CYP86B1*, which are crucial for incorporating aromatics into suberin, cutin and suberin-associated waxes, and TF MYB41, were defined as the direct targets of MYB49. Additionally, *MYB49* overexpressing lines demonstrate improved antioxidant defence (peroxidases and LEA) and elevated Ca^2+^ levels in leaves, thus enhancing salt tolerance in plants (Zhang et al., 2020).

WRKY family TF 33 was recently identified as a crucial regulator of salt tolerance in both Arabidopsis and rice. Mutants of *wrky33* and its direct target *atcyp94b1 (*involved in suberin biosynthesis) demonstrated reduced suberin content and salt-sensitive phenotype. However, *AtCYP94B1* expressing plants in *wrky33* background and rice wild-type improved both suberin content and salt tolerance, thus confirming the regulatory role of WRKY33 in CYP94B1-mediated salt-tolerance (Krishnamurthy et al., 2020). Similarly, AtWRKY9 was identified as a direct regulator of *AtCYP86B1* and *AtCYP94B3* responsible for salt stress tolerance. *Atwrky9* mutant had reduced roots suberin content and reduced expression of *AtCYP94B3* and *AtCYP86B1*. The salt-sensitive phenotype of *atcyp94b3* and *atcyp86b1* mutants was rescued by the expression of homologues genes from *Avicennia officinalis* (L.) *AoCYP94B3* and *AoCYP86B1* were also associated with reduced Na^+^ accumulation in the shoots (Krishnamurthy et al., 2021).

### 3.2 Suberin biosynthesis genes associated with plant salt tolerance

Suberin homeostasis is closely associated with fatty acid and carbohydrate metabolism. Similarly to other stress-related responses, ABA is involved in the suberin biosynthesis and deposition, CS formation and biosynthesis of compatible solute trehalose. However, the role of CS and CASPs family members requires further investigation.

Many individual genes involved in suberin biosynthesis have been demonstrated to improve the tolerance to salt stress. KCS (β-Ketoacyl-CoA synthase) catalyses the condensation of malonyl-CoA with acyl-CoA; thus, it is a crucial rate-limiting enzyme in the suberin biosynthesis (Rui et al., 2022). Expression of *KCS11* from grape (*Vitis vinifera* L.) in Arabidopsis increased its salt-stress tolerance on germination and seedling stages. This improvement was mediated *via* several mechanisms: accumulation of osmotic regulating substances (increased proline content), membrane stabilisation (reduced MDA content), and normalisation of ions homeostasis (increased expression of genes encoding several ion transporters and channels (such as *AKT1*, *CBLl9*, *CIPK23*, *SOS1*, *HKT1* and *CHX14* (Cation/H(^+^) antiporter)) (Yang et al., 2020). Similarly, expression of *KCS* from quinoa (*Chenopodium quinoa* Willd.) in Arabidopsis increased the occurrence of VLFAs (very long-chain fatty acids) with C22-24 chain lengths and promoting the accumulation of suberin monomers, thus improving salt tolerance (Tariq et al., 2022). These results demonstrated that suberin plays rather a universal role in salt tolerance and suggested *KCS* family members as potential genes to improve the salt-stress tolerance in different species.

Recently, the role of CASPs (Casparian strip membrane domain proteins) in salt tolerance was demonstrated. CASPs (UPF0497) are required for the CS formation at the endodermis and regulation of the selective uptake of mineral elements by roots, in particular Si and Ca^2+^ (Wang et al., 2019). The expression of *CASP4* from sorghum [*Sorghum bicolor* (L.) Mocnch] in Arabidopsis (in both WT and *atcasp5* mutant) increased salt stress tolerance, which was associated with lower level osmotic stress-associated damages and Na^+^ accumulation in leaves. Furthermore, transgenic plants had longer roots and higher expression of the genes related to Casparian strip formation (Wei et al., 2021). However, experiments of CS defective mutants suggested that CS formation and suberin deposition processes are interrelated and closely regulated by endogenous factors [small secretory peptides, such as CIF (Casparian strip Integrity Factor) and environmental factors (availability of nutrients, abiotic stresses)] (Doblas et al., 2017). Therefore, further research is required to better understand the complex association between CASPs, lignin, and suberin deposition under the influence of salt stress and the application of different regulatory proteins.

Additionally, TPS (trehalose-6-phosphate synthase), which is required for the biosynthesis of compatible solute trehalose, which is a crucial osmoprotector during salt and drought stresses (Nuccio et al., 2015), also mediated enhanced suberin-associated salt tolerance. In particular, rice *tps8* mutant demonstrated enhanced sensitivity to salt and ABA treatments, reduced content of soluble sugars, CS, and suberin deposition in the roots. In addition, ABA-responsive genes were down-regulated in *tps8* plants. On the contrary, *TPS8* overexpression rescued the salt-sensitivity associated phenotype and enhanced salt stress tolerance. These data suggested that rice TPS8 regulates salt tolerance *via* suberin deposition in an ABA-dependent way (Vishal et al., 2019). ABC family transporters (G type) are known to play a crucial role in suberin biosynthesis through the transport of suberin monomers from the cytoplasm to the apoplastic space (Shanmugarajah et al., 2019).

## 4 Conclusion and prospects

The cell wall is the first line of defence against environmental stresses. High salinity, as one of the worldwide distributed stress factors, can disrupt cell wall integrity and dysregulate normal signaling and nutritional functions. The severity of caused damage depends on salt concentration, availability of other ions (such as Ca^2+^ and K^+^) and associated stresses (drought, heat, and light intensity). Thanks to the great recent progress in our understanding of salt sensing and cell wall maintenance mechanisms under salt stress conditions, several new strategies for the improvement of the plant have been developed. In general, all kinds of plant modifications leading to the enforcement of physical cell wall barriers for salt transport, such as enhancement of suberisation and Casparian strip synthesis in root tissues, might greatly facilitate salt tolerance in plants. The development of gene editing technologies allows to generate of salt-tolerant varieties of cultivated species by target modification of the most relevant genes. First, the TFs—master regulators are responsible for cellulose, lignin, and suberin biosynthesis: HSFA7b, SND1, NAC83, MYB9, MYB41, MYB49, and WRKY33 (**Figures 3** and **5**). Increased production of ROS is a rather common and unspecific plant response to diverse stresses (including salt stress), thus suggesting antioxidants as an obvious and universal target to improve general plant tolerance to stresses. Members of the PME and XTH families respond to salt stress differently. However, overexpression or knockdown of specific PME or XTH genes may provide desirable outcomes: facilitate growth, yield and biomass accumulation, drought, and salt tolerance. Finally, despite multiple data supporting the role of abscisic acid in the salt stress-mediated modifications of the cell wall, our understanding of the role of plant hormones in the immediate and long-term cell wall modification pathways are rather limited and require further investigation.

In the past decades, extensive research has greatly improved our understanding of the mechanisms of salt stress tolerance in plants. However, the practical application of this vital knowledge to improve plant tolerance to salt stress is a rather slow and tardy process. The primary question in the field of salt stress response in plants is the identification of salt stress sensor/s. In this case it is necessary clearly define Na^+^/Cl^-^ sensors and sensors recognising osmotic, ionic and ROS-related signals. While certain progress has been made in the direction of plasma membrane salt stress sensing, Na^+^ perception if other organelles (ER, mitochondria, and chloroplasts) is much less exploited. In the next step, the integrating, coordinating and cross-talk mechanisms of salt-sensing signals from different organelles to provide optimised cellular response should be addressed. Also, it would be interesting to investigate the difference between Na^+^ and Cl^-^ sensors and signaling pathways in shoots and roots.

Moreover, salt-stress sensing and signaling in the cell wall relay and closely associated with Ca^2+^ channels, which need to be identified and characterised. Also, their connection to the cell wall repair mechanisms under salt stress conditions requires further investigation. Also, the circadian clock is known to regulate several crucial proteins (such as SOS1 antiporter) (Cha et al., 2022), thus adding another layer of complexity to the salt stress tolerance regulation. In this regard, it would be interesting to find out if other transporters and channels are subjected to circadian regulation and how they interact with cell wall biosynthesis processes. For example, how the SOS1-regulated pathway is related to the CesA internalisation, microtubule bundling and stability.

Currently, little is known about how the severity and duration of salt stress exposure correlate with cell wall changes and repair. While modern omics technologies can provide excess information about gene expression, protein, and metabolite abundance, the proper evaluation and establishment of regulatory mechanisms are usually missing. Furthermore, currently used methods of genetic engineering and biotechnology are based on single-gene analysis when the phenotype/genotype of every mutant (overexpressing or loss of function) is analysed independently. Such an approach is well-suited to study a particular pathway; however, it does not allow studying an organism as a system of interconnected and interdependent processes.

The expression of a specific gene in an organ-, tissue-, or cell-dependent context may provide additional complexity to the gene regulatory network at the whole-plant level. Also, while the root is the primary organ for salt sense and response, the molecular mechanisms regulating root-to-shoot signaling and transport need to be further elaborated. Finally, the role of known phytohormones and their cross-talk in the regulation of cell wall properties should be studied on the whole-plant level systemically.

## Author contributions

Conceptualization, methodology, formal analysis, SD and SI. Writing—original draft preparation, SD. Supervision, SI. Writing—review and editing, SD and SI. All authors contributed to the article and approved the submitted version.
